# Neurosphere-Derived Cells Exert a Neuroprotective Action by Changing the Ischemic Microenvironment

**DOI:** 10.1371/journal.pone.0000373

**Published:** 2007-04-18

**Authors:** Carmen Capone, Simona Frigerio, Stefano Fumagalli, Maurizio Gelati, Maria-Cristina Principato, Claudio Storini, Mery Montinaro, Rudolf Kraftsik, Marco De Curtis, Eugenio Parati, Maria-Grazia De Simoni

**Affiliations:** 1 Laboratory of Inflammation and Nervous System Diseases, Mario Negri Institute, Milano, Italy; 2 Laboratory of Neurobiology and Neuroregenerative Therapies, Carlo Besta Neurological Institute, Milano, Italy; 3 Department of Cell Biology and Morphology, University of Lausanne, Lausanne, Switzerland; 4 Clinical Epileptology and Experimental Neurophysiology Unit, Carlo Besta Neurological Institute, Milano, Italy; Centre de Regulacio Genomica - Barcelona Biomedical Research Park, Spain

## Abstract

**Background:**

Neurosphere-derived cells (NC), containing neural stem cells, various progenitors and more differentiated cells, were obtained from newborn C57/BL6 mice and infused in a murine model of focal ischemia with reperfusion to investigate if: 1) they decreased ischemic injury and restored brain function; 2) they induced changes in the environment in which they are infused; 3) changes in brain environment consequent to transient ischemia were relevant for NC action.

**Methodology/Principal Findings:**

NC were infused intracerebroventricularly 4 h or 7 d after 30 min middle cerebral artery occlusion. In ischemic mice receiving cells at 4 h, impairment of open field performance was significantly improved and neuronal loss significantly reduced 7–14 d after ischemia compared to controls and to ischemic mice receiving cells at 7 d. Infusion of murine foetal fibroblast in the same experimental conditions was not effective. Assessment of infused cell distribution revealed that they migrated from the ventricle to the parenchyma, progressively decreased in number but they were observable up to 14 d. In mice receiving NC at 7 d and in sham-operated mice, few cells could be observed only at 24 h, indicating that the survival of these cells in brain tissue relates to the ischemic environment. The mRNA expression of trophic factors such as Insulin Growth Factor-1, Vascular Endothelial Growth Factor-A, Transforming Growth Factor-β1, Brain Derived Neurotrophic Factor and Stromal Derived Factor−1α, as well as microglia/macrophage activation, increased 24 h after NC infusion in ischemic mice treated at 4 h compared to sham-operated and to mice receiving cells at 7 d.

**Conclusions/Significance:**

NC reduce functional impairment and neuronal damage after ischemia/reperfusion injury. Several lines of evidence indicate that the reciprocal interaction between NC and the ischemic environment is crucial for NC protective actions. Based on these results we propose that a bystander control of the ischemic environment may be the mechanism used by NC to rapidly restore acutely injured brain function.

## Introduction

Studies focusing on restoring injured brain function through cell transplantation have shown that cells infused by different routes or grafted into the brain may survive and lead to improvement of the damage observed in different brain disease models [1]–[4]. Cell replacement strategies are particularly promising candidate therapies for stroke, a condition in which a widespread loss of different cell types occurs that in principle may benefit from exogenous stem cells replacement of lost or damaged cells. However, while a number of reports show the effectiveness of this strategy, others fail to show significant results leaving many questions unresolved [3]–[5]. Successful reports, obtained using embryonic, bone marrow, umbilical cord or immortalized cell lines, such as NT2, demonstrated improvement of behavioral deficits determined by ischemia (reviewed in [3]–[5]). In spite of this evidence, the definition of the conditions for a successful treatment (the best source of stem cells, their optimal concentration, the best route of delivery, the degree of differentiation, *etc*…), as well as the mechanisms by which stem cells may exert their protective action are still largely unresolved. In principle, stem cells can act through several possible mechanisms, *i.e.* stimulating the plastic response or neural activity in the host damaged tissue, secreting survival-promoting neurotrophic factors, restoring synaptic transmitter release by providing local reinnervation, integrating into existing neural and synaptic network and reestablishing functional afferent and efferent connections. Presently, the lack of knowledge of the mechanisms driving stem cell protective action is a major obstacle to the advancement of cell replacement strategies in stroke research.

Neurospheres are free-floating heterogeneous aggregates that contain a minority of neural stem cells together with various progenitors and more differentiated cells. These clusters of cells, under appropriate culture condition, are able to produce neurons, astrocytes and oligodendrocytes [6], [7]. Neurosphere-derived cells (NC) have been successfully used in models of brain injury such as in experimental autoimmune encephalomyelitis or spinal cord injury [2], [6], [8].

In the present study we have used a murine model of focal, transient ischemia to investigate if 1) infused NC decrease ischemic injury and restore brain function; 2) these cells induce changes in the environment in which they are infused; 3) changes in brain environment consequent to transient ischemia are relevant for NC action.

## Results

**Figure 1:**
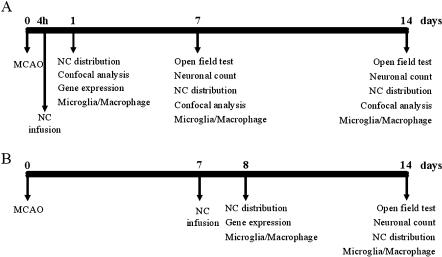
Experimental design. NC were infused icv either 4 h (A) or 7 d (B) after ischemia. Histology, immunohistochemistry, confocal microscopy, real time PCR, behavioural test and neuronal count experiments were performed at the time points indicated.

The experimental designs are illustrated in Fig. 1

### Open field test

**Figure 2:**
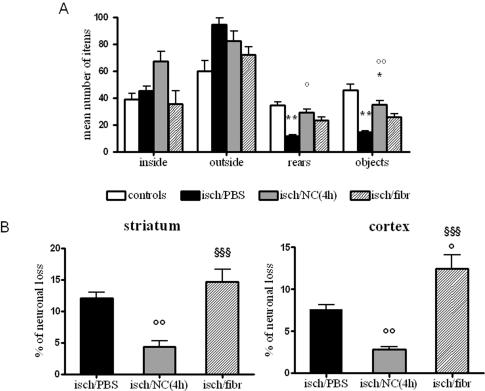
Open field test and neuronal loss in ischemic brain areas at day 7. Mice receiving NC(4 h) show a reversal of ischemia-induced impairment in number of rears and objects, (A, n = 10) and a significant reduction in neuronal loss in striatum and cortex (B) compared to those receiving PBS (n = 6). Fibroblast infusion 4 h after ischemia does not significantly affect open field impairment and neuronal loss (n = 7). Data are expressed as mean±SEM. One-way ANOVA: (A) rears F: 6.122, p = 0.0002; (A) objects F: 16.10, p<0.0001; (B) striatum F: 17.67, p = 0.0002; (B) cortex F: 37.01, p<0.0001. *Posthoc* Tukey test: °° p<0.01, °p<0.05 vs isch/PBS; ** p<0.01 *p<0.05 vs controls; §§§ p<0.001 vs isch/NC(4 h).

**Figure 3:**
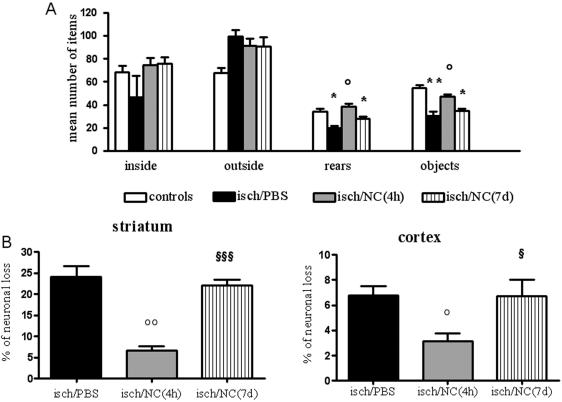
Open field test and neuronal loss in ischemic brain areas at day 14. Mice receiving NC(4 h), but not those receiving NC(7 d) show a reversal of ischemia-induced impairment in number of rears and objects (A, n = 10) and a significant reduction in neuronal loss in striatum and cortex (B) compared to those receiving PBS (n = 6). Data are expressed as mean±SEM. One-way ANOVA: (A) rears F: 11.46, p<0.0001; (A) objects F: 5.38, p = 0.0056; (B) striatum F: 31.67, p<0.0001; (B) cortex F: 5.06, p = 0.0256. *Posthoc* Tukey test: °° p<0.01, °p<0.05 vs isch/PBS, ** p<0.01, * p<0.05 vs controls; §§§ p<0.001, § p<0.05 vs isch/NC(4 h).

Seven days after ischemia mice receiving PBS icv (intracerebroventricular) injection showed a significantly lower number of rears and of contacts with objects indicating an impairment in exploratory behavior and sensory-motor activity respectively, with no change in inside (inversely related to anxious behaviour) and outside crossings (related to motor activity). This deficit was completely prevented in ischemic mice infused with NC 4 hours after ischemia (NC(4 h)), but not in those infused with fibroblasts that induced a non-significant improvement (Fig. 2A). In order to understand if NC protective effect was persistent, in a further experiment, ischemic mice receiving PBS or NC(4 h) were exposed to the same test 14 days after ischemia. Similarly to what observed at 1 week, ischemic mice infused with NC performed significantly better compared to those injected with PBS. At variance with these results, mice receiving NC 7 days after ischemia (NC(7 d)) did not show any recovery of these impaired functions (Fig. 3A)

### Neuronal loss

The same experimental groups of ischemic mice observed in the open field test were subsequently analyzed for the presence of neurodegeneration. Striatum, the brain area corresponding to the core of the lesion, and cortex, containing a portion of the lesion as well as the penumbra, were considered. Seven days after ischemia, neuronal loss (expressed as % of the contralateral) was 12.10±2.00 in striatum and 7.6±1.15 in cortex (black columns in Fig. 2B). In ischemic mice receiving NC(4 h) the neuronal loss was significantly reduced in both areas, being 4.14±2.46 (65.8% of reduction) and 2.84±0.83 (62.6% of reduction) in striatum and cortex, respectively (grey columns in Fig. 2B). Neuronal loss was not affected by fibroblast infusion (striped columns in Fig. 2B). We then evaluated the neuronal loss 14 days after the ischemia and found that the ischemic lesion was 24.19±5.85 in striatum and 6.29±1.88 in cortex of ischemic mice receiving PBS injections (black columns in Fig. 3B), while in those infused with NC(4 h) it was 6.70±2.56 (72.3% of reduction) and 3.12±1.45 (50.0% of reduction), in striatum and cortex respectively (grey columns). In mice receiving NC(7 d) the neuronal loss was not different from those injected with saline, being 22.11±2.91 in striatum and 6.72±2.85 in cortex (striped columns in Fig. 3B).

**Figure 4:**
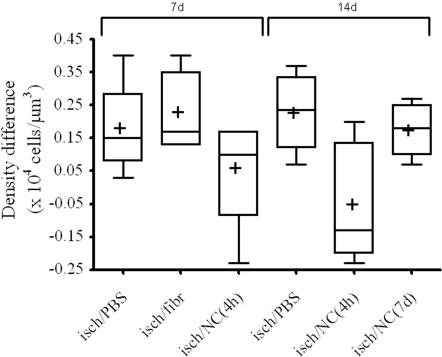
Box-plot of neuronal count performed in ischemic mice 7 and 14 days after ischemia with the optical dissector method. Differences of neuronal density between contralateral and ipsilateral striata are sorted by treatment and time. The box represents the median and 25^th^–75^th^ percentiles, whiskers represent the range, + represents the mean of the group. The hierarchical ANOVA showed a significant effect for the treatment (P = 0.02). The Bonferroni post-hoc test showed significant differences (P<0.05) among mean density differences of the isch/NC(4 h) group and the isch/PBS, isch/fibr and isch/NC(7 d) groups. Data plotted have been multiplied by 1×10^4^.

Since volume changes could have affected the neuronal count assessed by profile based method, a stereological assessment of neuronal density was also performed in striatum (Fig. 4). The hierarchical analysis of variance showed a significant effect for the treatment (P = 0.02). The time effect was not significant as was the interaction between time and treatment. The Bonferroni post-hoc test showed significant differences among mean density differences (and percentages) of the isch/NC(4 h) group and the isch/PBS, isch/fibr and isch/NC(7 d) groups.

### NC distribution and phenotype

**Figure 5:**
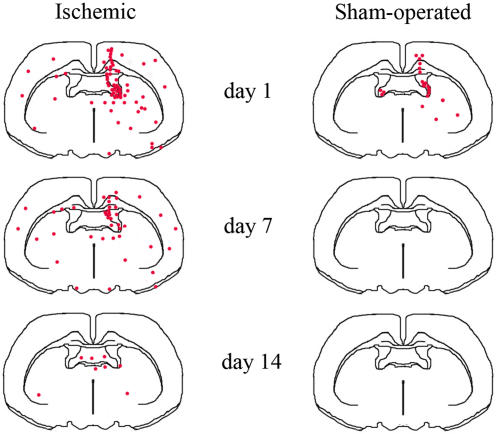
Distribution of NC(4 h) in ischemic and sham-operated mice at days 1, 7 and 14. NC were infused icv in the lesioned side. Drawings represent typical results and are based on n = 5 brains for each experimental condition.

**Figure 6:**
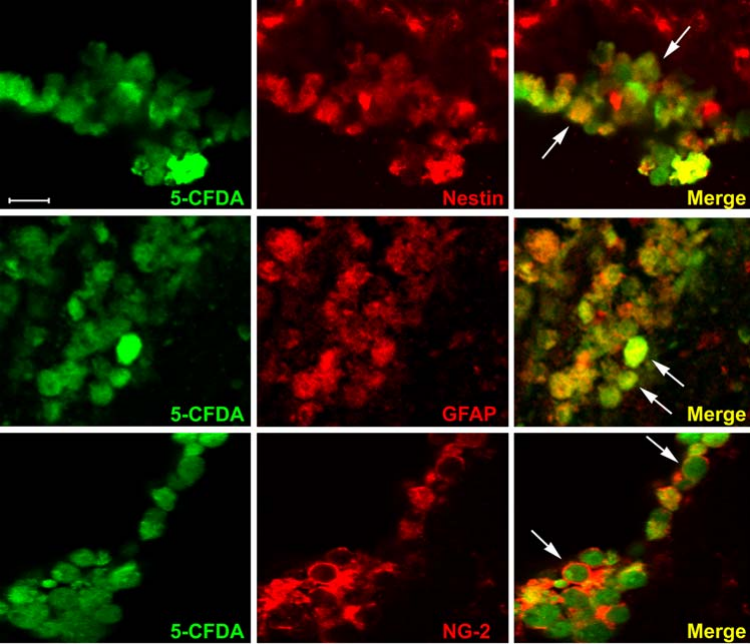
Confocal analysis of immunoreactivity of NC(4 h) - green - and nestin, GFAP or NG-2 - red - at day 1. Colocalization (arrows) can be observed between NC(4 h) and nestin, NC(4 h) and GFAP, NC(4 h) and NG-2. Images are taken in striatum, in proximity of the ventricular wall where most cells can be found at this time point. Bar: 25 µm.

We then analyzed NC presence and distribution in the brain of ischemic and sham-operated mice 1, 7 and 14 days after surgery (Fig. 5). One day after ischemia and NC(4 h) infusion (left panel in Fig. 5), 5-CFDA (5-carboxy-di-O-acetylfluorescein) positive cells were mostly located in the injected right ventricle (ipsilateral to the lesion) and a few of them could be found in the left ventricle. Some NC were juxtaposed to the ventricular walls both in the right and in the left hemisphere, and some were already diffusing from the ventricles into the striatum. A few NC were located along the icv injection route. At day 7 the general distribution of NC was similar to that observed at day 1 but the total number of NC was lower. Fluorescent cells could be found heterogeneously dispersed in ipsi- and contra- lateral brain areas. At day 14 only a few cells were still present and could be observed in the ventral hippocampal commissure (Fig. 5). At variance with ischemic, in sham-operated mice few NC were present only at day 1, while at days 7 and 14 no cells could be found in any of the 5 brains per time point analyzed (right panel in Fig. 5). Similarly to what observed in sham-operated mice, in ischemic mice infused with NC(7 d) only few cells could be observed 1 day after infusion (day 8 from ischemia) and no cells could be found at day 14 (n = 6). As mentioned above, all the data on NC distribution have been obtained with 5-CFDA fluorescent cells. Additionally, few mice per group have been infused with Hoechst labeled NC, completely confirming the results described above. Immunohistochemical evaluation demonstrated that both at day 1 (Fig. 6) and at day 7 (data not shown), most NC(4 h) were positive for nestin (a marker of neural stem cells) and GFAP (Glial Fibrillary Acidic Protein, a marker for astrocytes) and a few for NG-2 (a marker for oligodendrocyte precursors). At day 14 neither nestin+, GFAP+ nor NG-2+ cell could be observed (data not shown).

### Gene expression

**Figure 7:**
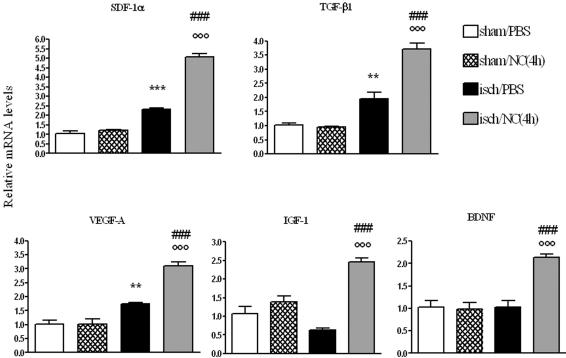
mRNA expression of cytokines and trophic factors in ischemic cortex at day 1. Data are expressed as fold of induction compared to sham/PBS group (mean±SEM, n = 6). Two-way ANOVA: SDF-1α F: 97.52, p<0.0001; TGF-β1 F: 31.54, p = 0.0001; VEGF-A F: 24.14, p = 0.0004; IGF-1 F: 28.21, p = 0.0002; BDNF F: 19.34, p = 0.0009. *Posthoc* Bonferroni test: **p<0.01, ***p<0.001 vs sham/PBS; °°°p<0.001 vs isch/PBS; ### p<0.001 vs sham/NC(4 h).

In order to evaluate possible changes induced in the ischemic environment by NC infusion, at day 1 we analyzed the mRNA expression of a few genes in the cortex of ischemic and sham-operated mice infused with NC(4 h) (Fig. 7). We first analyzed the chemokine Stromal Derived Factor-1α (SDF-1α) and found that ischemia significantly increased its expression. In ischemic mice infused with NC its expression was further enhanced. No change was observed in sham-operated mice infused with NC compared with those infused with PBS. A similar pattern of expression was found for the cytokine TGF-β1 and for the Vascular Endothelial Growth Factor A (VEGF-A). In both cases ischemia activated their expression and infusion of NC to ischemic mice was able to elicit a higher increase. Ischemia induced a slight, non significant decrease in the expression of Insulin Growth Factor-1 (IGF-1), but ischemic mice receiving cell infusion showed a clear cut induction of this gene. Lastly, Brain Derived Neurotrophic Factor (BDNF) was significantly increased in ischemic mice infused with NC with respect to controls (Fig. 7). A similar analysis of the same genes was done in mice receiving NC(7 d), one day after NC infusion (8 days after ischemia, see experimental design in Fig. 1B). None of these genes was significantly activated in any experimental group.

### Microglia/macrophages

**Figure 8:**
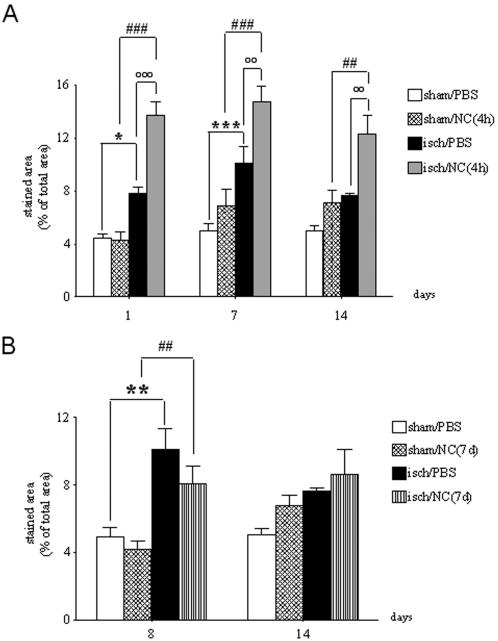
Quantification of microglia/macrophage activation. The area occupied by CD11b immunopositive cells was measured in striatum of sham-operated or ischemic mice receiving PBS, NC(4 h) or NC(7 d) at different time points (see Experimental design, Fig. 1A and B). Data are expressed as mean±SEM (n = 4–5). Two-way ANOVA: (A) F: 58.05, p<0.0001; (B) F: 10.19, p = 0.0002. *Posthoc* Bonferroni test: *p<0.05, **p<0.01, ***p<0.001 vs sham/PBS; °°p<0.01, °°°p<0.001 vs isch/PBS, ##p<0.01, ###p<0.001 vs sham/NC.

**Figure 9:**
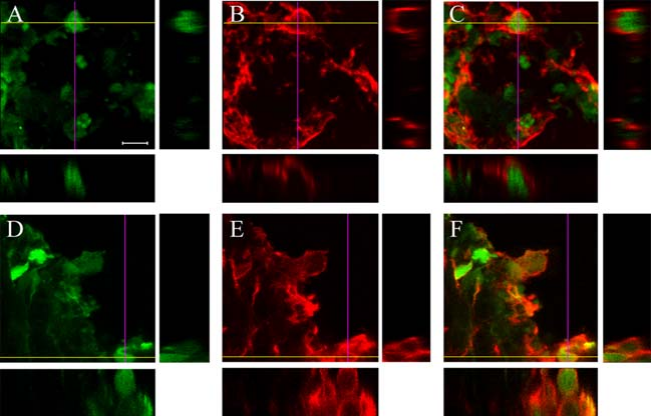
Confocal analysis of immunoreactivity of NC (green) and microglia/macrophages (red). CD11b immunoreactivity signal, staining for microglia/macrophages, colocalizes with that of NC(4 h) in striatum at day 1 (A–C) and 7 (D–F). Z- stacks are shown. Bar: 25 µm.

**Figure 10:**
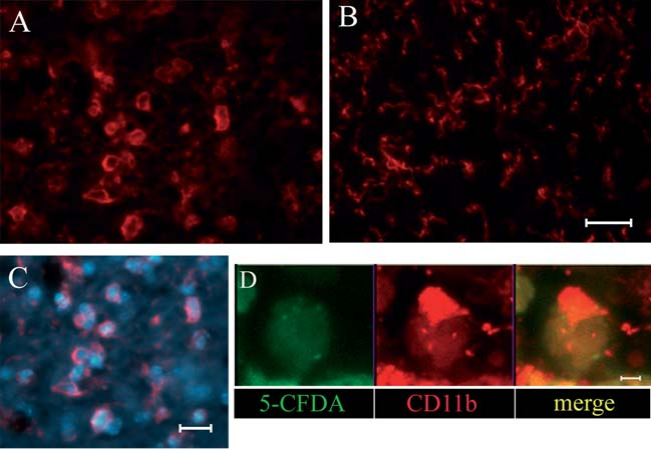
Morphology of microglia/macrophages (red). CD11b immunopositive cells at day 1 after ischemia and NC(4 h) infusion display a round, phagocytic morphology in the injected (A) but not in the contralateral side were no NC are present (B). Microglia/macrophages appear to surround NC as evidenced in C were NC are marked with Hoechst (blue). A further detail of the interaction between these cells is provided in D where one microglia/macrophage appears to cap a NC (marked with 5-CFDA, green). Bar (A–C): 20 µm; bar (D): 7 µm.

Available antibodies (anti-CD11b) do not distinguish between resident activated microglial cells and recruited macrophages, thus the results obtained with immunohistochemistry provide information on the degree of activation/recruitment of both these inflammatory cells [9]. As expected, microglia/macrophages became activated in ischemic mice. The maximal expression was reached at day 7 (Fig. 8A). At each time point analyzed, in ischemic mice receiving NC(4 h), the area occupied by CD11b immunopositive cells measured in striatum was further increased. Notably, such activation was neither present in sham-operated mice infused with NC (Fig. 8A and B) nor in ischemic mice infused with NC(7 d) (Fig. 8B). We then analyzed the interaction of microglia/macrophages with the infused cells by confocal microscopy. A high degree of colocalization of the markers for both NC and microglia/macrophages was apparent at day 1 as well as day 7 (Fig. 9). To get an insight into the functional meaning of microglia/macrophage activation we explored their morphology in more detail. We found that CD11b immunopositive cells had a round shape exclusively in the ischemic area containing the infused cells (Fig. 10A). CD11b^+^ microglia/macrophages appeared to surround NC (Fig. 10C), and indeed were found to cap them when observed at higher magnification (Fig. 10D). In the same sections where this morphology was apparent, but in an area not containing NC, CD11b^+^ cells showed a ramified morphology with thin cell bodies, indicating that they were not activated (Fig. 10B).

## Discussion

### Effectiveness of NC infusion

The first major finding of this study is the observation that NC(4 h) revert functional deficits and counteract neurodegeneration in ischemic mice. As early as one week after ischemia, the impairment in exploratory behavior and sensory-motor activity observed in ischemic mice was no more apparent in ischemic mice receiving NC(4 h). This protective effect was observed also at day 14, as assessed in independent experimental groups of mice. Also the evaluation of the neuronal loss, both in striatum and in cortex, confirmed the marked protective effect of NC. At both time points, on day 7 and day 14, the neuronal cell count showed that ischemic mice receiving NC(4 h) infusion were significantly protected. Although several studies have addressed the issue of cell replacement therapy in ischemic injury [3]–[5], to our best knowledge this is the first study to show functional recovery and reversal of neurodegeneration by NC in ischemic injury.The choice of NC has been a successful one. The infused cells include different stages of multipotent stem cells. In specific *in vitro* conditions they are able to differentiate into astrocytes, neurons and oligodendrocytes. Following infusion in the brain, at day 1 and day 7, we observed that most NC maintained their undifferentiated features being mostly nestin+ and GFAP+, therefore indicating an abundance of immature neurons and astrocytes, a few of them expressing markers of oligodendrocyte precursors (NG-2+). In view of the early recovery observed, we can exclude that NC(4 h) protective effect was due to cell differentiation or replacement. We can hypothesize that their protective action was due to a bystander mechanism. The experiments discussed below elucidate their possible protective mechanism.

### Distribution and survival of NC in ischemic brain tissue

We chose icv delivery of cells since it is less invasive, allows to deliver more cells and produces more extensive cell seeding than the local intraparenchymal injection. Moreover, compared to systemic injections, icv delivery allows to infuse a lower number of cells that are not dispersed in the whole body, but they are instead concentrated where they are needed.From the injected ventricle, NC rapidly diffused and migrated to several brain areas. At each time point studied (days 1, 7, 14) NC(4 h) were mostly found in the injected side, *i.e.* ipsilateral to the lesion. The comparison between NC(4 h) distribution at different time points in ischemic and sham-operated mice indicated that the ischemic environment affected their survival in the brain tissue. In sham-operated mice, indeed, the infused cells did not diffuse in the parenchyma and were rapidly cleared out. Thus either molecules acting as attractant for these cells or inhibiting their disposal are produced by the ischemic environment. Similarly to what happened in sham-operated mice, in ischemic mice infused with NC(7 d) the infused cells did not persist in the brain tissue, but they were instead rapidly eliminated, indicating that at this time point the ischemic environment has lost its ability to promote NC survival. Another important issue derives from the observation that even in ischemic mice NC were progressively lost. It has been shown that the survival of stem cells in brain tissue depends on several factors, including the type of cell infused and its degree of differentiation. In agreement with our observations, it was shown that multipotent neural stem cells, (differently from lineage-restricted neural precursors) do not survive in the intact CNS [10]. We show here that NC that do not survive in sham-operated animals, can instead survive in the ischemic brain tissue, although for a limited time. Notably this period of time is long enough for them to exert a protective effect, a condition that is almost ideal.

### Gene expression

To explore the mechanism through which NC promote recovery and to analyze the changes in the ischemic environment, we assessed the mRNA expression of some protective factors. We analyzed Stromal Derived Factor 1α (SDF-1α), a chemokine produced by glial and endothelial cells in the injured brain that binds the CXCR4 receptor expressed on NC and enhances their proliferation and transmigration [11]–[14]. We then analyzed TGF-β1, a pleiotropic cytokine involved in cell growth, differentiation and tissue repair whose expression is induced in ischemic brain. Its protective effects have been associated with ischemic tolerance [15]–[17]. We also evaluated VEGF-A mRNA expression, an angiogenic factor that undergoes transcriptional and posttranscriptional induction by hypoxia. It has been shown to exert acute neuroprotective effects and to stimulate neurogenesis in ischemic brain [18]–[20]. A specific role in promoting the survival of selected NC subpopulation has recently been shown [21]. We then analyzed IGF-1 a neurotrophic and anti-apoptotic factor known to activate the survival PI3K-Akt pathways [22], [23]. It is induced in the injured brain although transient decrease in mRNA expression, similar to what we observed, has also been reported [24]. Its expression is required for brain vascular remodeling [25]. In a recent study IGF-1 has been identified as one of the upregulated diffusible factors involved in promoting neural progenitor proliferation after focal ischemia [26]. Finally, we assessed the expression of BDNF, a neurotrophic factor provided with multiple effects on brain plasticity that regulates neuronal survival, cell migration and synaptic function [20], [27]. The expression of all these factors at day 1 was significantly enhanced in ischemic mice receiving NC(4 h) compared to those receiving PBS. Thus activation of all these trophic factors occurs maximally when NC are infused into the ischemic brain. Notably, neither cell infusion in sham-operated mice, nor that at day 7 from ischemia (NC(7 d)) elicited this response. Thus the reciprocal interaction between NC and ischemic environment is crucial for the protective actions of these cells.

### Microglia/macrophages

Due to the lack of cellular markers that discriminate between resident microglia and recruited macrophages, in this study we refer to microglia/macrophages to designate cells immunoreactive for CD11b, although most of them are probably likely to be typical resident microglia. In a recent study on green fluorescent protein transgenic bone marrow chimeric mice it was shown that the vast majority of immunocompetent cells (iba-1+) present in the brain tissue after transient middle cerebral artery occlusion (MCAO) derives from resident microglia and that this cell type markedly predominates and anticipates hematogeneous macrophages [28]. Our data show that NC(4 h) trigger an important activation of microglia/macrophages, selectively in the ischemic tissue. Together with recruited macrophages, resident microglial cells are the major effectors of the brain immune response. During the process of activation induced by a variety of stimuli (such as ischemia) microglia proliferates, acquires dramatic changes in morphology, expresses novel surface antigens and produces mediators that build up and maintain the inflammatory response of the brain tissue [29], [30]. While microglia produces inflammatory cytokines, chemokines, toxic molecules such as nitric oxide synthase, arachidonic acid, reactive oxygen species and complement proteins that contribute to the ischemic cell death, considerable evidence now shows that resident microglia may also produce anti-inflammatory and protective molecules, growth factors and neurotrophins, indicating that its activation may result in either toxic or trofic effects [29], [30]. Moreover, recent data show that distinct subpopulations of microglial cells are activated by a CNS injury [31]. These cellular subsets express selected antigens that may subserve different roles including repair and recovery from injury. Our data strongly suggest that activation of microglia/macrophages is required for NC action. In fact NC(4 h), but not NC(7 d) or NC infused to sham-operated mice, are effective in protecting from ischemic injury and induce a more marked microglia/macrophage activation. Consistent with this observation, previous data in the literature indicate that the brain inflammatory response is relevant for stem cell survival and action [3], [8], [32].Our data also show other aspects of the complex interaction between NC and microglia/macrophages that appear to be engaged in a close physical reciprocal contact. This interaction resembled what has been previously described as “capping” of the NC by amoeboid microglia [33]. It is possible that this cell-cell contact is related to microglia/macrophage phagocytic activity. If this is the case, it could be speculated that the progressive disappearance of NC from the brain from day 1 to 14 results from such phagocytic action. Thus, microglia-induced cell death in the early hours that follow brain ischemia may be regarded as a protective mechanism against further secondary damage that could “prime” the damaged regions for later regeneration [34].Several *in vitro* studies have shown that microglia does not constitute a uniform population but it rather includes different cell types that can develop a braod range of functional phenotypes [35]. Our *in vivo* data support this view indicating that ischemia-activated microglia can on one hand contribute to rescuing the damaged tissue. We can speculate that microglia may trigger or contribute to the production of trophic factors and protective cytokines. On the other hand microglia may get rid of infused cells by a typical phagocytic acitivity implying a NC-microglia cell contact.

### Conclusions

NC, administered 4 h after transient focal ischemia, effectively counteracted the ischemia/reperfusion injury in an *in vivo* animal model of MCAO. Namely NC reverted both functional impairments evaluated by testing for exploratory behavior and sensory-motor activity, and decreased neuronal loss. Several lines of evidence indicate that the reciprocal interaction between NC and the ischemic environment is crucial for NC protective actions. First, NC survival in the brain tissue is enhanced by the ischemic environment. Second, NC induce the activation of chemokine, angiogenic and neurotrophic factor transcripts early after injury. Third, NC protective effect relates to microglia/macrophages activation. Thus, NC can promote protective effects in ischemia/reperfusion injury by modifying the ischemic environment via induction of trophic factors and activation of selected aspects of the inflammatory response. Based on these results we propose that a bystander control of the ischemic environment may be the mechanism used by NC to rapidly restore acutely injured brain function.

## Materials and Methods

### Animals

Male C57BL/6 (26–28 g, 11–12 weeks of age, Charles River, Calco, Italy) were housed 5 per cage and kept at constant temperature (21±1°C) and relative humidity (60%) with regular light/dark schedule (7 *am*–7 *pm*). Food (Altromin pellets for mice) and water were available *ad libitum*. Procedures involving animals and their care were conducted in conformity with institutional guidelines that are in compliance with national (D.L. n.116, G.U. suppl. 40, 18 February 1992) and international laws and policies for animal care (EEC Council Directive 86/609, OJ L 358,1; Dec.12, 1987; NIH Guide for the Care and Use of Laboratory Animals, U.S. National Research Council 1996).

### Isolation and culture of NC

As previously described [6], [7], [36], [37], brains of newborn mice were removed and placed in PBS containing penicillin and streptomycin (GIBCO, Paisley, Scotland, UK). Brain tissue was mechanically triturated with a fire-polished Pasteur pipette. The tissue suspension was collected and treated with collagenase type I (0.5% BSA, 0.1% collagenase in PBS w/o Ca^2+^ and Mg^2+^, GIBCO, Paisley, Scotland, UK) and DNAse Type I (1 U/mL, USBiological/DBA, Segrate, Italy) for 30–45 minutes at 37°C in a humidified atmosphere containing 5% CO_2_. After centrifugation, pellets were plated in culture flasks in the presence of 20 ng/ml human recombinant EGF and bFGF in basal serum-free medium optimized for neural stem cell growth [6], [36]. Neurospheres formed within 7 days were harvested and dissociated to single cells with collagenase and replated twice under the same conditions described above. Bulk cultures were generated by passing the cells at higher density in the same growth medium every 10 days. Cells for transplantations were used starting from 7 passages. After dissociation, cell count and viability test were assessed by trypan blue exclusion.

### Foetal fibroblast culture

A commercially available cell line of murine foetal fibroblasts (NIH 3T3) was used as negative control since they are not derived from CNS. Fibroblasts were cultured in Dulbecco's modified Eagle's medium with 4 mM L-glutamine containing 1.5 g/L sodium bicarbonate and 4.5 g/L glucose and 10% of bovine calf serum (all Gibco, OR, USA) at 37°C degrees and 5% CO_2_ in an humidified atmosphere. When the cell layer reached the 80% of confluence, cells were washed with PBS and 2.0 to 3.0 ml of Trypsin-EDTA solution was added to each flask. Cells were observed under an inverted microscope until cell layer was dispersed. Complete growth medium (6.0 to 8.0 ml) was added in order to block the trypsin–EDTA effects and cells were aspirated by gently pipetting. After a brief centrifugation cells were resuspended in 3–5 ml of PBS with Dnase.

### 5-CFDA and Hoechst staining

Before infusion, NC were labelled with fluorescent dyes for in vivo tracing. Cells were exposed to 7.5 µM green fluorescent dye 5-CFDA (Vybrant SE Cell Tracer kit V-12883, Molecular Probes, Eugene, OR, USA) for 15 min at 37°C in a humidified atmosphere containing 5% CO_2_ and then resuspended in PBS without calcium and magnesium. Alternatively, they were stained with 10 µg/µL Hoechst 33258 (2,3 bis-benzimide, Sigma, Mo, USA) for 1 h at 37°C in their culture medium and subsequently resuspended in PBS without calcium and magnesium. In both cases, the absolute number of cells was evaluated by light microscopy. Vitality of NC after fluorescent staining was evaluated in each experiment by trypan blue exclusion test.

### Transient focal cerebral ischemia

Ischemia was achieved by intraluminal model of MCAO as previously described [9], [38], [39]. To confirm the adequacy of the vascular occlusion in each animal, blood flow was measured by laser Doppler flowmetry (Transonic BLF-21) using a flexible 0.5 mm fiberoptic probe (Transonic, Type M, 0.5 mm diameter) positioned on the brain surface in the MCA territory and secured with impression material on the skull at the following stereotaxic coordinates: AP = −1 mm; L = −3.5 mm. The filament was advanced in the MCA until a >70% reduction of pre-ischemic blood flow was observed. At the end of a 30 min ischemic period, blood flow was restored by carefully removing the nylon filament. During surgery mice were maintained at constant temperature (37+/−0.5°C) using a heathing pad equipped with a rectal probe (LSI-Letica Spain). Sham-operated mice were also prepared as previously described [9], [38], [39].

### NC and fibroblast administration

NC (5×10^5^ in PBS) were infused icv either 4 hours (NC(4 h)) or 7 days (NC(7 d)) after the end of MCAO (Fig. 1) in a volume of 5 µl over 10 minutes, using an automatic micropump (CMA, Carnegie Medicine, Sweden) in anesthetized mice (0 mm caudal to bregma, −1 mm lateral to the midline and −3 mm beneath the *dura mater*) [40]. The needle was left in place afterwards for additional 5 minutes. Fibroblasts (5×10^5^ in PBS) were infused 4 hours after the end of MCAO following the same procedure.

### Open field test

Behavior was evaluated in an open field paradigm [41], [42], delivered once to each mouse, 7 or 14 days after ischemia/reperfusion. During 5 min observation the number of inner squares crossed and that of outer squares crossed, the number of rearings, except those for grooming, and the number of contacts with the objects were counted by an investigator blinded to the treatment.

### Neuronal count: profile based method

Brain sections obtained from perfused brains were mounted on slides and were stained with Cresyl Violet. Neurons were counted as described previously [43]. Briefly, for each mouse three coronal sections, corresponding to +0.30, −0.30 and −0.70 mm from bregma, were considered and neurons in striatum and in cortex were counted separately. An Olympus BX61 microscope, interfaced with Soft Imaging System Colorview video camera and AnalySIS software (all Olympus Tokyo, Japan) was used. The degree of neuronal loss was calculated by pooling the number of stained neurons in the sections of each hemisphere and was expressed as percentage of the contralateral hemisphere. The analysis was performed by an investigator blinded to the treatment.

In a previous study we have shown that the lesion protocol used results in a neuronal loss of 14.0+/−2.2% measured in striatum at day 7 and corresponds to a lesion volume of 12.11+/−1.44 mm^3^ measured at day 1. Actually it should be noted that seven days after ischemia the lesioned area cannot be precisely assessed by the paleness of the tissue following neutral red, cresyl violet or similar staining because of the large microglial activation and cell recruitment to the lesion site. This is why at this time point, the ischemic lesion is assessed by neuronal count [17].

### Stereological estimation of the neuronal density

Neurodegeneration in striatum was assessed also by the optical dissector method using the StereoInvestigator program (Microbrightfield Inc.) and the Zeiss Imager microscope with a motorized XY stage and focus. The image of the field view was transferred on the computer monitor using an Optronics Magnifire camera. For each striatum 3 sections chosen systematically (coordinates: +0.30, −0.30 and −0.70 mm from bregma, respectively) were used to assess the non biased density. The section contour was digitized using a cursor at low magnification (2.5×) and was fractionated in counting frames of 250×300 µm. At high magnification (100×) boxes of 50×50×20 µm were used to count the neurons inside each frame. The frames were scanned systematically under program supervision starting with a randomly chosen frame. The neurons present in the top reference section and absent in the bottom lookup section were counted as tops. More than 500 tops were counted for each striatum. The density was estimated from the total number of tops counted in the striatum divided by the total volume of counting boxes.

The statistical analysis was carried out using the SAS software package (SAS Institute Inc., Cary, NC, USA). The distribution of measurements was tested for normality. The general linear model (GLM) procedure was applied to test the treatment (the following groups were considered: isch/PBS, isch/fibr, isch/NC(4 h), isch/NC(7 d)) and time (7 d and 14 d) effect, using a hierarchical two way analysis of variance model. Then a post-hoc Bonferroni test was used to assess the difference between groups mean at P<0.05.

### Immunohistochemistry

Immunohistochemistry was performed applying standard protocols. The primary antibodies used were: monoclonal anti-GFAP (1∶2000), anti-Nestin (1∶1000), anti-NG-2 (1∶200) (all antibodies from Chemicon International, CA), anti-CD11b (1∶300, a kind gift of Dr. A. Doni, Mario Negri Institute, Milan, Italy). The secondary antibodies were: Alexa 555 fluoro-conjugated goat anti-mouse IgG for nestin and GFAP or Alexa 594 fluoro-conjugated goat anti-rat IgG for CD11b and goat anti-rabbit IgG for NG-2 (all Molecular Probes, OR). Microglia/macrophages activation was revealed by reaction with 3,3 diaminobenzidine tetrahydrochloride (DAB, Vector laboratories, CA) as previously described [43]. Immunopositive cells were evaluated in striatum by an image analysis software (AnalySIS, Olympus Tokyo, Japan). For each mouse, 3 sections, corresponding to coordinates −1.10 mm, −0.70 mm and −0.20 mm from bregma, were considered. In each slice, 6 fields at 20× were analyzed. Results are expressed as ratio between immunostained area and the total area of the fields assessed (100%).

### Confocal analysis

Immunofluorescent staining was acquired by an Olympus BX61 microscope equipped with an Olympus confocal scan unit FV500 equipped with two laser lines, Ar-Kr green (488 nm) and He-Ne red (646 nm) used to detect the NC marker, 5-CFDA, and secondary antibodies conjugated at Alexa 555, respectively. Colocalization was revealed using a scanning sequential mode to eliminate possible bleed-through effect. The system was managed by Fluoview software (Olympus Tokyo, Japan)

### RNA isolation, cDNA synthesis and real-time PCR

At day 1 or 8 mice were sacrificed for gene expression analysis. Brains were rapidly removed and lesioned side cortex dissected and frozen on dry ice. Total RNA was obtained from tissue specimen using the Trizol reagent (Gibco BRL, MD) [9], [44]. Samples of total RNA (1.5 µg) were reverse transcripted with random hexamer primers using Multi-Scribe Reverse Transcriptase (TaqMan Reverse transcription reagents, Applied Biosystems, Foster City, CA). Real-time PCR was conducted according to manifacturer instructions [9], [44]. β-actin was used as reference gene and relative gene expression levels were determined according to manufacturer's ΔΔCt method (Applied Biosystems). Primers were designed using Primer Express 2.0 software (Applied Biosystems, Foster City, CA) based on GenBank accession numbers (Table 1).

**Table 1:** Primers used for quantitative real-time polymerase chain reaction analysis of SDF-1α, BDNF, IGF-1, VEGF-A, TGF-β1, β-actin transcript levels.

cDNA	Sequence (5′ to 3′)	Amplified region
SDF-1α	GATTGTTGCACGGCTGAAGA (fwd^a^)	255–305
NM013655	TTCGGGTCAATGCACACTTG (rev^b^)	
BDNF	AGGCACTGGAACTCGCAATG (fwd^a^)	1271–1321
NM007540	AAGGGCCCGAACATACGATT (rev^b^)	
IGF-1	TGGAGATGTACTGTGCCCCA (fwd^a^)	1609–1659
NM184052	GGATAGAGCGGGCTGCTTTT (rev^b^)	
VEGF-A	CCTGCAAAAACACAGACTCGC (fwd^a^)	882–932
NM009505	CGTTTAACTCAAGCTGCCTCG (rev^b^)	
TGF-β1	CCAAGGAGACGGAATACAGGG (fwd^a^)	1494–1544
NM011577	TCACAAGAGCAGTGAGCGCT (rev^b^)	
β-actin	GCCCTGAGGCTCTTTTCCAG (fwd^a^)	850–900
NM007393	TGCCACAGGATTCCATACCC (rev^b^)	

a forward

b reverse
